# e-Estesia: A Serious Game for Reducing Arousal, Improving Emotional Regulation and Increasing Wellbeing in Individuals with Gambling Disorder

**DOI:** 10.3390/jcm11226798

**Published:** 2022-11-17

**Authors:** Teresa Mena-Moreno, Lucero Munguía, Roser Granero, Ignacio Lucas, Fernando Fernández-Aranda, Mónica Gómez-Peña, Laura Moragas, Antonio Verdejo-García, José M. Menchón, Susana Jiménez-Murcia

**Affiliations:** 1 Department of Psychiatry, University Hospital of Bellvitge, Bellvitge Institute for Biomedical Research (IDIBELL), 08907 Barcelona, Spain; 2 Ciber Fisiopatología Obesidad y Nutrición (CIBERObn), Instituto de Salud Carlos III, 28029 Madrid, Spain; 3 Department of Psychobiology and Methodology, Autonomous University of Barcelona, 08193 Barcelona, Spain; 4 Department of Clinical Sciences, School of Medicine and Health Sciences, University of Barcelona, 08034 Barcelona, Spain; 5 School of Psychological Sciences, Turner Institute for Brain and Mental Health, Monash University, Clayton, VIC 3800, Australia; 6 Ciber Salut Mental (CIBERSAM), Instituto de Salud Carlos III, 28029 Madrid, Spain

**Keywords:** treatment outcomes, emotion regulation, complementary intervention, serious games, gambling disorder

## Abstract

Gambling disorder (GD) is associated with deficits in emotion regulation and impulsivity-related personality traits. In recent years there has been an increase in the use of serious games (SG) to address these factors with positive results. The aim of this study was to analyze the efficacy of the intervention with a new SG (e-Estesia), as an adjunct to a CBT intervention for GD. The sample comprised two groups (experimental group (*n* = 40) and control group (*n* = 64)) of patients with GD diagnosis. Both groups received 16 weekly CBT sessions and, concurrently, only the experimental group received 15 additional sessions with e-Estesia. Pre-post treatment with e-Estesia administered in both groups were: DSM-5 Criteria, South Oaks Gambling Screen, Symptom Checklist-Revised and measure of relapses, dropout and compliance of treatment. As regards the experimental group were also administered: Difficulties in Emotion Regulation Scale, Emotion Regulation Questionnaire, and Impulsive Behavior Scale. No statistically significant differences in the general psychopathological state, emotion regulation or impulsivity were found when comparing the groups. However, patients enrolled in the e-Estesia intervention had significantly less relapses and better indicators of treatment compliance than the control group. Considering these results, the use of complementary tools such as SG are useful for addressing GD.

## 1. Introduction

Gambling Disorder (GD) is characterized by a persistent and recurrent gambling behavior, regardless of the negative consequences for the individual’s life (family, professional or personal areas. GD is considered a multicausal behavioral addiction in which different psychological, biological and environmental factors intervene and interact [1]. Both difficulties in emotion regulation [2,3,4,5] and high impulsivity have been associated with severity in GD [6,7,8]. Several studies show a strong association between impulsivity and problematic gambling behavior [9,10]. What is more, impulsivity is a risk factor for GD [11,12,13,14,15,16,17,18].

Impulsivity can be defined as the predisposition to carry out behaviors without premeditation and to react prematurely to stimuli. It is a multifactorial construct, which can be divided into five dimensions according to the model of Lynam et al. (2006) [19]. The validity of this model has been established through several studies using the UPPS-P scale [20], which measures the five dimensions [19,21]: (i) lack of premeditation; (ii) lack of perseverance; (iii) sensation seeking; (iv) positive urgency; (v) negative urgency.

In terms of Emotion Regulation (ER), it is about managing both the emotional process and the experience of different emotions in diverse settings. ER is a complex process, essential for psychological wellbeing and for establishing appropriate relationships with other people. Gratz & Roemer (2004) [22] classify difficulties in emotion regulation into six dimensions: non-acceptance, goals, impulse, awareness, strategies and clarity.

For the last two decades, the process of regulating emotional responses in stressful situations has been increasingly incorporated into the understanding of psychopathology development [23,24,25,26]. Different studies show individual differences in emotion regulation as important risk or protective factors for psychopathology, as well as the use of one type or another of emotion regulation strategies [23,27]. In this vein, gambling could be used as a strategy to escape from problems and negative emotional states [28,29]. It can serve as a maladaptive mechanism for regulating positive (e.g., empowerment, euphoria) and negative (e.g., boredom, sadness or stress) emotions [30]. This also accords with other studies, which showed that, GD patients reported greater ER impairments than individuals without this disorder, as well as a greater lack of emotional clarity and emotional awareness, and less use of reappraisal as an adaptive emotional regulation strategy or a counterproductive use of this strategy [2,4,31]. It has been described that ER is strongly related to the psychopathological state of GD patients [32].

The ability to regulate emotions is a factor that influences the way people respond to and recover from stress [25]. Stress is frequently associated with negative emotions and can become a risk factor for the development of different psychopathologies if not approached in a healthy way [33,34]. The physiological response to stress is characterized by the activation of the sympathetic nervous system (SNS), that involves the secretion of (nor)adrenaline that produces a rapid increase in the heart rate, respiratory frequency and blood pressure [35]. Heart rate variability (HRV), an index of consecutive changes in the heartbeats [36] and reflects the flexible regulation of autonomic arousal according to the demands of the environment [37]. It is considered a promising biomarker of adaptive ER [38].

At rest, a higher HRV is associated with better regulation of negative affect (downward) and a more flexible emotional response and the use of adaptive emotion regulatory strategies [38]. Inasmuch as the HRV measurement reflects the autonomic flexibility, an increase in HRV correlates with greater emotional control [37]. The management of physiological variables (such as HRV) to handle stressful situations and achieve better emotion regulation has been studied through the use of relaxation techniques such as slow breathing [39,40,41,42]. Studies such as that of Jerath et al. (2015) [43] have shown that paced breathing [44] is associated with states of relaxation and well-being, while fast breathing is often associated with states of anxiety and stress [45]. Different studies with a non-clinical sample show the results of the use of breathing management and the use of biofeedback with measures of HRV to reduce psychological stress (intrusive thoughts, anxiety or fear of losing control), reduce anxiety and make more habitual use of coping strategies adaptive emotion regulation [46,47,48].

These positive effects of breath management led to respiratory training applications [49,50,51,52] and games that use breathing control to help people reach a relaxed state [52,53,54]. Some applications also use biofeedback, which promotes the effectiveness of breathing training [55]. Biofeedback is a technique in which individuals learn to adapt their behaviors based on physiological cues (e.g., HRV).

In recent years, there has been an increase in the use of applications and video games as a complementary tool for mental health treatments (i.e., serious games) [56,57,58,59,60,61,62,63,64,65,66]. In a review, it is concluded that different interventions using serious games (SG) showed positive results on psychotherapy, and were particularly effective in increasing motivation, enhancing adherence to treatment and strengthening the therapeutic alliance [67]. There are various studies on the impact of psychological interventions on GD, such as motivational interviewing, self-help interventions, psychodynamic interventions or cognitive behavioral therapy (CBT) [68,69]. CBT is the most widely used and has strong evidence supporting its efficacy [68,70]. It reduces global severity, frequency of gambling, and financial losses effectively [71], and is particularly effective when it includes motivational components [72]. Cognitive restructuring [73,74], a fundamental part of this therapy, affects cognitive factors associated with gambling, such as irrational beliefs and magical thinking, and promotes patients’ understanding of cognitive distortions related to gambling behavior [75,76]. However, although therapies based on cognitive-behavioral approaches are considered an effective treatment for GD [70,71,77], the dropout and relapse rates are quite high when treating these patients. Dropout occurs when an individual discontinues treatment before its completion. The premature dropout rate in people with GD undergoing CBT is around 30–50% [78,79,80]. This ratio is troubling, since the treatment outcomes for these patients will be worse [81,82]. The use of technology-based complementary methods could reduce the high rates of drop-out in GD. In this vein, a study found that patients using SG had a lower dropout rate than patients not using them. In conclusions, complementing cognitive-behavioral therapy (CBT) interventions in GD with a serious video game might be helpful in addressing certain underlying factors which are usually difficult to change, such as impulsivity or anger expression [58]. Self-training via SGs in GD patients with biofeedback sensors has been shown to reduce general impulsive behaviors and arousal, as well as enhancing self-control [58,61,64]. Another recent study, combining a serious virtual game with traditional therapy (CBT) for GD patients, finds positive results [83].

In recent years, video games have ceased to be considered mere entertainment tools and are used to offer interactive and complex narratives that can be used for therapeutic purposes. An SG is a custom-made video game, specifically designed to achieve predefined objectives related to educational purposes or skills training in the most enjoyable or entertaining way possible [65]. Recent systematic reviews in the context of SGs report that health is the second most frequent domain for the use of video games, the first being learning [56,84,85].

The proliferation of SGs in the context of mental health occurs for several reasons. They often facilitate participation and motivation to continue in the therapeutic process. Likewise, it has been shown that immersive experiences at a sensory level (at a visual, auditory or tactile level) improve the effectiveness of these therapeutic tools. In addition, they can be attractive in recruiting people who might not otherwise seek treatment. Finally, they can be easily accessible and affordable for those who have financial limitations to obtain professional help [86,87].

SGs allow the participants to recreate real situations in virtual scenarios while provoking a series of cognitive, emotional and behavioral responses. These responses allow them to train specific skills in a motivating and entertaining way, an objective that can be more difficult to achieve with traditional therapies. In general, some studies have reported that participants prefer SG interventions to standard interventions, finding that SGs are able to address ER deficits effectively [88].

The aims of this study were, on the one hand, to analyze if the use of e-Estesia could help improve the treatment adherence, treatment outcomes and psychopathological state of GD patients that are receiving CBT and, on the other hand, to analyze the efficacy of e-Estesia in improving emotion regulation abilities and impulsivity. Both groups receive the usual cognitive-behavioral treatment for this disorder, but in the so-called experimental group a complementary intervention with the SG has also been carried out. It is hypothesized that the experimental group will have better treatment outcomes: lower relapse rates, lower dropouts and better compliance with cognitive-behavioral treatment than control group, as an indicator of better adherence to treatment. Additionally, it is expected a better psychopathological state at the end of treatment in these patients than the group that did not receive this complementary intervention with e-Estesia.

## 2. Materials and Methods

### 2.1. Participants

The sample consisted of 104 patients voluntarily seeking treatment for GD. They were divided into two groups (experimental group versus control group). Experimental group consisted of 40 patients with GD diagnosis while the control group consisted of 64. Patients were randomly assigned to each group. Data from participants were collected from February 2018 to November 2019.

The inclusion criteria for selecting the subjects was the presence of GD diagnosis, while the exclusion criteria were: (a) the presence of a psychiatric or neurological disorder (e.g., schizophrenia), (b) an intellectual disability and (c) active pharmacological therapy that might interfere with game performance.

### 2.2. Procedure

Therapy intervention consisted of 16 group weekly CBT sessions and, concurrently, for the experimental group, 15 additional sessions with the SG were added. The 16 intervention sessions (CBT) are described in the manual protocol of the Bellvitge University Hospital. After providing informed and signed consent, participants were scheduled for an individual appointment in a week’s time. In the experimental group, this appointment was utilized to (a) brief the participants about the study procedure; (b) administer the pre-treatment questionnaires (c) explain how to use the device and sensor strap; (d) train participants how to ‘beat’ the app (i.e., breathing calmly, regulating one’s emotions, etc.), and (e) conduct an initial supervised session using e-Estesia. After conducting the initial intervention session with the application, patients were instructed to use the SG at home for the next 13 days, always at the same time. The last session (session 15) was held in the hospital (at the Department of Psychiatry), then post-treatment questionnaires with e-Estesia were administered.

**Figure 1:**
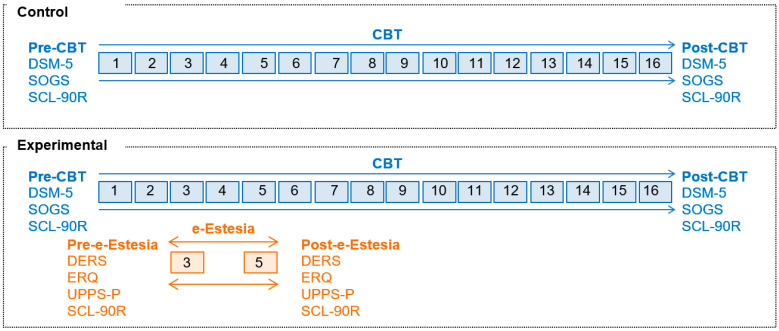
Scheme with the design of the study. Note. CBT: Cognitive behavioral therapy. DERS: Difficulties in Emotion Regulation Scale. ERQ: Emotion Regulation Questionnaire. UPPS-P: Impulsive. Behavior Scale. SCL-90R: Symptom Checklist-Revised. SOGS: South Oaks Gambling Severity Screen. DSM-5: number of DSM-5 criteria for gambling disorder.

The pre-post treatment with e-Estesia questionnaires administered to patients in the experimental group were: DSM-5 Criteria, South Oaks Gambling Screen (SOGS), Symptom Checklist-Revised (SCL-90-R), Difficulties in Emotion Regulation Scale (DERS), Emotion Regulation Questionnaire (ERQ), and Impulsive Behavior Scale (UPPS-P). In the control group, only these pre-post treatment with e-Estesia questionnaires were administered: DSM-5 Criteria, South Oaks Gambling Screen (SOGS) and Symptom Checklist-Revised (SCL-90-R) (see Figure 1).

All participants received information regarding the aims of the research, and they provided signed informed consent for participating. There was no financial or other compensation for being part of the study. Participants who agreed to take part in the research were briefed on the purpose of the study and were reassured of the voluntary nature of their participation and their rights to stop at any time. The study was approved by the Ethics Committee of the first author’s hospital (ref. number PR286/14), adhering to the principles outlined in the latest version of the Declaration of Helsinki.

### 2.3. Measures

#### 2.3.1. Diagnostic Questionnaire for Pathological Gambling (According to the Diagnostic and Statistical Manual of Mental Disorders [DSM] Criteria) [89]

This diagnostic questionnaire allows us to assess the presence of GD through 19-items based on the DSM taxonomy [for the DSM-5 versions [90]. Spanish Adaptation reached good psychometric properties, Cronbach alpha α = 0.81 calculated for the general population and α = 0.77 for a clinical sample. Patients were assessed through a face-to-face clinical interviews performed by a trained clinician [91].

#### 2.3.2. South Oaks Gambling Severity Screen (SOGS) [92]

This self-report questionnaire screens gambling-related problems through 20 items and provides a total score which is used as a measure of gambling severity. Very good psychometric properties (test–retest reliability R = 0.98, internal consistency α = 0.94 and convergent validity R = 0.92) are shown by the Spanish validation of the scale’s questionnaire [93]. The internal consistency in the study sample was in the good range (α = 0.843).

#### 2.3.3. Symptom Checklist-Revised (SCL-90-R) [94]

This self-report tool measures the global psychological state through 90 items structured in nine primary dimensions (somatization, obsessive–compulsive, anxiety, hostility, phobic anxiety, depression, interpersonal sensitivity, paranoid ideation and psychoticism) and three global indices (GSI: global severity index; PST: positive symptoms total; and PSDI: positive symptoms discomfort index). The GSI is the indicator of the current level of severity of perceived malaise. This questionnaire has obtained good to adequate indices (mean α = 0.75) for the Spanish version [95].

#### 2.3.4. Difficulties in Emotion Regulation Scale (DERS; [22])

This self-report questionnaire contains 36 items that assess difficulties in regulating emotions. It is consists of six scales: (1) non-acceptance of emotional responses; (2) difficulties in engaging in goal-directed behavior; (3) impulse-control difficulties; (4) lack of emotional awareness; (5) limited access to effective ER strategies; (6) lack of emotional clarity. It also contains a global measure (total scale). The Spanish version has demonstrated adequate psychometric features [96], which has been used in this study.

#### 2.3.5. Emotion Regulation Questionnaire, Spanish Version (ERQ; [97])

It is a 10-item questionnaire to assess the respondents’ tendency to implement two ER strategies: reappraisal and emotional suppression. Validity and internal consistency are adequate (Cronbach’s α = 0.75, 0.71, respectively).

#### 2.3.6. Impulsive Behavior Scale (UPPS-P)

Fifty-nine items measure five facets of impulsive behavior: negative urgency; positive urgency; lack of premeditation; lack of perseverance; and sensation-seeking [21]. It has shown good reliability (Cronbach’s α between 0.79 and 0.93) and external validity [98] in the Spanish-language adaptation.

#### 2.3.7. Measure of Dropout, Relapses and Compliance

Dropout: interruption of the cognitive-behavioral treatment before its completion.

Relapse: lack of abstinence from gambling during cognitive behavioral treatment. It was defined as an isolated episode of gambling associated with mild negative consequences on the patients’ economy and family.

Treatment compliance: parameter established by the group therapist based on whether the patient completes the weekly records at home (e.g., money spent on each activity); depending on whether the patient meets the guidelines indicated session by session or not (level of participation in the sessions).

#### 2.3.8. Sociodemographic and Clinical Variables

**Figure 2:**
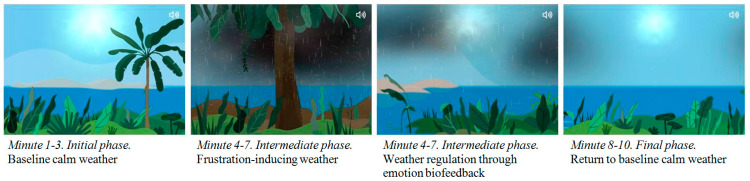
Animation sequence of the application e-Estesia.

Semi-structured interviews were conducted in order to registered additional sociodemographic features, such as sex, education level, and marital and employment status. Likewise, we assessed the socioeconomic position according to Hollingshead’s index (which provides a global measurement based on the participants’ profession and level of education) and other gambling problem-related variables (age of onset of the gambling behaviors, duration of gambling activity).

e-Estesia: a serious game for reducing arousal, improving ER and increasing wellbeing:

e-Estesia is an app-based SG designed to reduce arousal, improve ER and increase wellbeing. It was inspired by a previous SG named PlayMancer [62,64]. e-Estesia, keeps the benefits of Playmancer, including the biofeedback through a HR and HRV sensor, but allows patients to use the device at home, after an initial diaphragmatic breathing training and the first session monitored by a specialist. The game has an island landscape, where the gamer’s point of view is located a few feet inland and moves alongside the coastline. Each session lasts 10 min. During the first 3 min, the landscape is sunny and the patient is invited by a voice in off to maintain a relaxing state and breathe deeply using diaphragmatic breath. In the following 4 min, rain clouds and eventually a storm will appear, and the patient would be able to handle the landscape through the biofeedback: if the patient remains calm, the rain and the clouds start to disappear, but, if the patient do not manage to maintain a calm state, the storm gets stronger. The final 3 min are aimed to keep calm and relax as the first 3 min (Figure 2 shows an animation sequence). To date, e-Estesia runs on password-protected Android portable devices, connected via Bluetooth to the biosensor. More information about the usability of the SG can be found in the article by [99].

### 2.4. Statistical Analysis

Data were analyzed with Stata17 for Windows (Stata Corporation, College Station, TX, USA). The baseline comparisons between the control and experimental groups were done with *t*-tests for independent groups for quantitative variables, and with chi-square tests (χ^2^) for categorical variables. The comparison between the pre- post-changes in the GD severity and the psychopathology state after the CBT was tested with 2 × 2 mixed analysis of variance (ANOVA). These procedures defined the group (control versus experimental) as the between-subjects factor and the measure (pre versus post) as the within-subjects factor. The changes pre and post the use of the e-Estesia registered among the experimental group were assessed with ANOVA for related samples. For all these procedures, partial-eta^2^ coefficients estimated the effect size of each contrast obtained in the ANOVA procedures (values of 0.06, 0.10, and 0.25 were interpreted as low-poor, moderate-medium, and large-high effect size [100]), and Cramer’s-V values the effect size for the results obtained in the χ^2^ tests procedures (values of 0.10, 0.20, and 0.30 were interpreted as low-poor, moderate-medium, and large-high effect size [101]). In addition, the Finner’s-method was used to control the increase in the Type-I error due to multiple comparisons (this method is a Familywise error rate stepwise procedure with more statistical power than the classical Bonferroni correction [102]).

Kaplan-Meier function estimated the cumulative survival curve for the rate of dropout and relapse during the CBT, and Log-Rank tests compared the survival curves between the groups. The cumulative survival function is used to measure the probability of patients “living” (surviving without the presence of the outcome, in this study dropout or the presence of relapse episodes) for a certain amount of time after the beginning of the intervention. One advantage of this procedure is allowing modeling censored data, which occurs if patients withdraw from the study (arrive alive to the end of the follow-up or are lost in the follow-up without event occurrence at last measurement time).

Receiver Operator Curve (ROC) was used to assess the optimal cutoff of the e-Estesia for the presence of relapses. The identification of the cutoff was based on the prevalence of relapse-episodes estimated in the sample of the study.

## 3. Results

### 3.1. Characteristics of the Sample

Most participants in the study were men (95.2%), with primary education level (51.9%), single (49.0%) or married (41.3%), employed (63.5%) and within mean-low to low social position indexes (78.8%). Mean age was 40.2 years-old (SD = 14.7), mean onset of the gambling problems 30.0 years (SD = 11.7) and mean duration of the disorder 5.3 years (SD = 5.8). Table S1 (Supplementary Materials) contains the description and the comparison between the groups (statistical differences only were found for the employment status, with higher proportion of unemployed participants among the experimental group).

Table 1 includes the correlation matrix with the most relevant variables of the study, measured at the pre-treatment and post-intervention. Relevant correlation was considered for coefficients with effect size within the moderate (|*R*| > 0.24) to large (|*R*| > 0.37). At the pre-treatment the next associations were found: (a) the number of DSM-5 criteria for GD positively correlated with the other measures (except for the ERQ reappraisal scale), and the SOGS total score correlated with impulsivity and the psychology distress; (b) the emotion dysregulation measured with the DERS correlated with the impulsiveness and the psychology distress levels; and (c) higher impulsivity also correlated with worse psychology state. After the use of the app, the same pattern of associations was found.

### 3.2. Analysis of the Pre-Post Changes after the Use of the e-Estesia (Experimental Group, n = 40)

Table S2 (Supplementary Materials) contains the changes reported before and after the use of the e-Estesia. Significant decreases were observed for the ERQ suppression scale and the psychopathology state (SCL-90R scales, except for the phobic anxiety).

### 3.3. Analysis of the CBT Outcomes

Table 2 contains the results of the 2 × 2 mixed ANOVA with the pre-post comparison of the GD severity and the psychopathology state. All the interaction parameters group-by-time are in the non-significance zone (*p* > 0.10), suggesting that differences in the different conditions are statistically equal. However, since our aim was also to estimate the effect size of the differences, single effects for the factors group and time were estimated and interpreted. No differences between the groups were observed at baseline (pre-CBT). However, the experimental group obtained lower mean scores (compared to the control group) in the SCL-90R phobic anxiety and psychotic scales. Significant pre-post decreases were registered for both groups in all the scales analyzed in Table 2.

**Figure 3:**
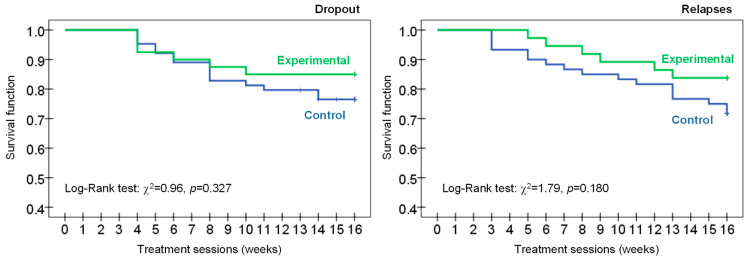
Survival analysis for the rate of dropout and relapses (Kaplan-Meier function) (total sample, *n* = 104).

Table 3 includes the analysis of dropouts, relapses and compliance with the guidelines during the CBT (see also Figure 3, with the survival functions for the rate of dropout and relapse). No differences between the groups were obtained for the risk of dropout and the number of sessions attended. However, the patients in the experimental condition achieved lower likelihood of relapses and higher likelihood of good compliance with the treatment rules.

### 3.4. ROC Study for the Use of the e-Estesia

**Figure 4:**
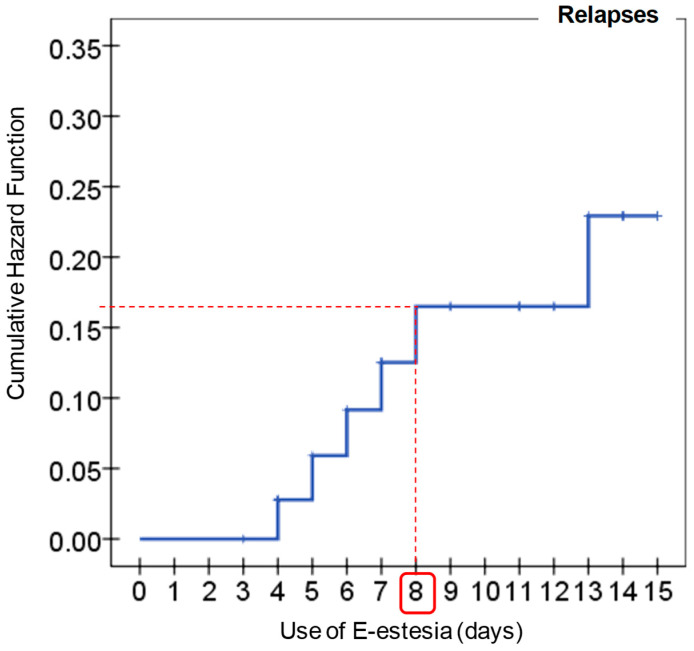
Survival analyses for the rate of relapses (experimental group, *n* = 40). Note. Cumulative Hazard Function: one minus Kaplan-Meier cumulative function.

Figure 4 contains the survival analysis for the rate of relapses among the experimental group. The X-axis shows the number of days e-Estesia was used and the Y-axis shows the cumulative hazard function (one minus the Kaplan-Meier function). According to the survival curve, the optimal cutoff point for the use of the App was 8 days (most of the relapses were registered prior to this use).

Figure S1 (Supplementary Materials) includes a new figure that analyzes the capacity of the e-Estesia to discriminate the risk of relapses. In this figure, the X-axis displays the number of days using the App, and the three plotted lines represent the Sensibility (Se), the Specificity (Sp) and the False Alarm Rate (FAR, defined as one minus the Sp). The cutoff of 8 days using e-Estesia obtained Se = 0.833 (83.3%), Sp = 0.667 (67.7%) and the area under the ROC curve was 0.728. Figure S2 (Supplementary Materials) includes the predictive capacity of e-Estesia for different prevalences of relapses (X-axis). The different plotted lines represent the predictive value for positive (PV+), the predictive value for negative (PV−), and the False Discovery Rate (FDR, defined as one minus the PV+).

## 4. Discussion

The aims of the present study were to analyze the efficacy of the intervention using e-Estesia, comparing the treatment results between experimental (CBT + e-Estesia) and control (CBT) groups of GD patients. The results obtained show that both therapy approaches, CBT and CBT + e-Estesia, are effective for the intervention in GD. Both groups present statistically significant lower scores in GD severity and better general psychopathological state at the end of CBT. These findings were expected to be that this intervention has been mentioned as the most efficient for GD [70,71,77,103].

Focusing on the comparison of both groups, there were no statistically significant differences in the general psychopathological state (except for the subscales of phobic anxiety and psychotic), in which significantly lower scores were found in the experimental group). The e-Estesia group had less relapses and lower indicators of bad compliance of treatment than the control group, which could be interpreted along the lines that the experimental group showed better adherence to the treatment guidelines. The results are in agreement with the previous literature, which reports that interventions using SGs have been proven to be helpful to increase engagement in the treatment of different psychiatric disorders such as depression, obsessive compulsive disorder, eating disorders, and even GD [58,104,105,106].

One of the benefits of the use of SGs in mental health is the improvement of the adherence to the treatment, by increasing the motivation, helping to develop positive relationships between patients and therapists and reducing the dropouts rates [58,67,107,108]. The SGs are a valuable part of modern psychotherapy, having advantages as not tied to specific therapy times. The patient decides the time, place and pace of learning, increases autonomy and the feeling of self-control in the therapeutic process and strengthens the active role of the patient in therapy [109]. In addition, SGs generate a sense of challenge, curiosity, control and choice, factors that can affect motivation and commitment to therapy [110,111,112]. Due to the profile of GD patients, this type of complementary interventions could be especially useful, taking into account the characteristics or personality traits that these types of patients usually present: cognitive and emotional impulsivity, and preference for immediate versus delayed rewards [7,113,114,115,116,117,118,119,120]. What is more, these characteristics could make adherence to treatment more difficult in the medium and long term [121,122,123,124,125]. Previous studies in GD patients demonstrated an existing relationship between lack of perseverance and treatment dropout as well as negative urgency and number of relapses during treatment [121]. A study concluded that positive urgency predicts an increased probability of drop-out and that low levels of sensation seeking together with greater awareness of the gambling problem predict a higher degree of adherence to therapy (enhanced compliance with therapeutic guidelines and instructions) [120]. In light of the results of this study, e-Estesia has been shown to be as effective as other SGs in improving adherence to treatment. These patients had less relapses and lower indicators of bad compliance of treatment than the control group.

The experimental group showed no reductions in impulsivity levels or in the emotion regulation. Considering that impulsivity is a personality trait and as such, generally stable over time, it is not surprising that no differences were obtained when comparing pre- and post-intervention levels with e-Estesia.

In addition, in terms of emotion regulation strategies it has been found a significant reduction in the use of the suppression as an emotion regulation strategy in the group of patients who received the intervention with e-Estesia. Emotional suppression (vs. emotional reappraisal) is a maladaptive emotion regulation strategy, which is often used by GD patients [126,127]. This result is in the same direction as research which demonstrates that the use of breathing management and biofeedback with measures of HRV could contribute to generate an habitual usage of adaptive emotion regulation and coping strategies [47]. Finally emotional distress levels present a significant decrease, except for the phobic anxiety subscale. In this vein, the use of the app also has a positive impact in the general psychopathology of the GD patients. It seems that phobic anxiety is not sensitive to the intervention with e-Estesia (at least not immediately), but it is to the complete intervention with e-Estesia + CBT compared to the group that only receives CBT, improving at the end of all the sessions. Other investigations such as that of Tárrega et al. [58] also show that complementary interventions to CBT with a serious game can improve the psychopathological state of the patient, showing a reduction in all scores (including phobic anxiety). In view of the results, it could be hypothesized that the use of e-Estesia improves well-being, as well as a relaxed and mindful state, positive effects that have been found with the respiratory training and the management of self-regulation through peace breathing [43,49,50,51,52,53].

Considering these positive results of the use of the app and exploring relapses from another perspective, specifically what is the minimum use of the app to reduce the number of relapses, findings show that 8 sessions with the app would be the optimal required to stop relapses. With this final analysis, not only are we presenting results that support that the app can be an important complementary tool for the treatment of GD, but we have also been able to identify which is the optimal therapeutic indication for the reduction of relapses in GD patients.

The results of this study should be considered in light of its limitations. First, in the sample, most of the participants were male (only 5 women participated). Some studies carried out in healthy participants have found gender-related differences in some variables such as the response impulsivity [128] among others. Our study included all patients who sequentially were attended for treatment due to the GD related problems and met inclusion criteria, and since in the clinical area the proportion of men is larger than the proportion of women, the female sex was underrepresented in the work. However, we considered it relevant to keep the female subsample to provide greater ecological validity to the research. Future studies would benefit from including women and comparing both groups and thus be able to extrapolate the results to the population. Second, it has not been possible to compare the results of trait impulsivity (UPPS-P) and emotion regulation (DERS; ERQ) between the experimental group and the control group, only an intragroup comparison has been made regarding these measures in the experimental group. It would be interesting for future studies to compare these variables between both groups.

In future studies it is proposed that a greater number of sessions are carried out with this SG to be able to observe if the treatment results can improve exponentially.

## 5. Conclusions

Although there are effective treatments to address behavioral addictions such as GD, the use of complementary tools such as SG are useful for addressing these disorders. In this study, e-Estesia has improved adherence to treatment (fewer relapses and adherence to therapeutic guidelines) of GD patients. The evaluation, registration, and treatment tools based on applications for tablets and mobile phones allow dynamic actions, in real time, known as Ecological Momentary Interventions (EMI), with the aim of collecting data as accurately as possible, while giving the support that each patient needs.

## Figures and Tables

**Table 1:** Correlation matrix for the main variables of the study.

		1	2	3	4	5	6	7
1	DSM 5 criteria	---	**0.589** **^†^**	**0.342** **^†^**	**0.252** **^†^**	0.208	**0.378** **^†^**	**0.349** **^†^**
2	SOGS total	**0.834** **^†^**	---	0.023	0.016	0.091	**0.310** **^†^**	**0.363** **^†^**
3	DERS Total score	**0.314** **^†^**	**0.284** **^†^**	---	**0.407** **^†^**	0.054	**0.411** **^†^**	**0.377** **^†^**
4	ERQ Suppression	**0.302** **^†^**	**0.268** **^†^**	**0.431** **^†^**	---	**0.350** **^†^**	0.180	0.213
5	ERQ Reappraisal	0.062	-0.028	0.099	**0.307** **^†^**	---	0.223	0.190
6	UPPS-P Total score	0.162	0.030	**0.603** **^†^**	**0.405** **^†^**	0.026	---	**0.312** **^†^**
7	SCL-90-R GSI	0.034	0.127	**0.472** **^†^**	**0.433** **^†^**	0.019	**0.289** **^†^**	---

Note. Upper part of the table: correlation coefficients at pre-treatment (total sample, *n* = 104). Down part of the table: correlation coefficients at post-treatment (experimental group, *n* = 40). ^†^ Bold: effect size within the ranges moderate to large.

**Table 2:** Pre-post changes registered for the CBT: 2 × 2 mixed ANOVA.

	Control (CO)				Experimental (EXP)					Contrasts									
	(*n* = 64)				(*n* = 40)				Inter.	Factor Group (CO-EXP)				Factor Time (Pre-Post)					
	Pre-CBT		Post-CBT		Pre-CBT		Post-CBT		Group	Pre-CBT		Post-CBT		Control			Experimental		
	Mean	SD	Mean	SD	Mean	SD	Mean	SD	Time	*p*	η^2^	*p*	η^2^	*p*	η^2^	Power	*p*	η^2^	Power
GD severity																			
DSM 5 criteria	7.39	1.39	2.13	2.04	7.10	1.75	1.93	2.13	0.848	0.350	0.009	0.634	0.002	0.001 *	**0.761 ^†^**	1.000	0.001 *	**0.658 ^†^**	1.000
SOGS total	11.1	3.14	6.11	3.85	11.1	3.32	4.78	3.17	0.152	0.946	0.000	0.069	0.032	0.001*	**0.442 ^†^**	1.000	0.001*	**0.440 ^†^**	1.000
Psyc. (SCL-90R)																			
Somatization	0.91	0.79	0.48	0.42	0.88	0.82	0.33	0.40	0.863	0.482	0.005	0.132	0.022	0.001 *	**0.209 ^†^**	0.999	0.001 *	**0.155 ^†^**	0.990
Obsess-comp.	1.16	0.85	0.61	0.51	0.94	0.63	0.44	0.37	0.565	0.703	0.001	0.083	0.029	0.001 *	**0.327 ^†^**	1.000	0.001 *	**0.280 ^†^**	1.000
Sensitivity	1.02	0.87	0.47	0.54	0.90	0.78	0.32	0.39	0.863	0.482	0.005	0.132	0.022	0.001 *	**0.209 ^†^**	0.999	0.001 *	**0.155 ^†^**	0.990
Depression	1.54	1.03	0.67	0.60	1.46	0.96	0.48	0.45	0.565	0.703	0.001	0.083	0.029	0.001 *	**0.327 ^†^**	1.000	0.001 *	**0.280 ^†^**	1.000
Anxiety	1.04	0.79	0.44	0.46	0.94	0.83	0.32	0.42	0.914	0.522	0.004	0.177	0.018	0.001 *	**0.250 ^†^**	1.000	0.001 *	**0.181 ^†^**	0.997
Hostility	0.89	0.86	0.45	0.49	0.71	0.75	0.37	0.60	0.597	0.278	0.012	0.434	0.006	0.001 *	**0.132 ^†^**	0.974	0.016 *	0.055	0.676
Phobic anxiety	0.42	0.60	0.17	0.23	0.30	0.38	0.09	0.11	0.723	0.279	0.011	0.047 *	0.038	0.001 *	**0.124 ^†^**	0.965	0.012 *	0.060	0.716
Paranoia	0.94	0.78	0.43	0.42	0.79	0.78	0.37	0.38	0.589	0.355	0.008	0.459	0.005	0.001 *	**0.212 ^†^**	0.999	0.001 *	**0.104 ^†^**	0.927
Psychotic	0.92	0.77	0.37	0.37	0.88	0.76	0.20	0.22	0.434	0.757	0.001	0.012 *	0.061	0.001 *	**0.260 ^†^**	1.000	0.001 *	**0.244 ^†^**	1.000
GSI	1.07	0.74	0.50	0.45	0.96	0.67	0.35	0.33	0.771	0.461	0.005	0.073	0.031	0.001 *	**0.274 ^†^**	1.000	0.001 *	**0.214 ^†^**	0.999
PST	46.9	22.0	25.1	17.6	44.8	20.2	20.9	13.5	0.653	0.620	0.002	0.197	0.016	0.001 *	**0.362 ^†^**	1.000	0.001 *	**0.298 ^†^**	1.000
PSDI	1.85	0.59	1.41	0.38	1.76	0.60	1.25	0.50	0.612	0.450	0.006	0.065	0.033	0.001 *	**0.205 ^†^**	0.999	0.001 *	**0.179 ^†^**	0.997

Note. SD: standard deviation. Inter: interaction group-by-time. η^2^: Partial eta-squared. Observed power (calculated for alpha = 0.05). * Bold: significant comparison. ^†^ Bold: effect size within the ranges moderate to large.

**Table 3:** CBT outcomes: dropout, relapses and compliance with the CBT rules.

	Control *n* = 64		Experimental *n* = 40				
	*n*	%	*n*	%	*p*	C-V	Power
Risk of dropout							
Yes	15	23.4%	6	15.0%	0.297	0.102	0.186
No	49	76.6%	34	85.0%			
Risk of relapses							
Yes	17	28.3%	6	16.2%	0.173	**0.** **138 ^†^**	0.160
No	43	71.7%	31	83.8%			
Compliance with rules							
Bad	22	36.1%	5	13.5%	0.015 *	**0.** **245 ^†^**	0.779
Good	39	63.9%	32	86.5%			
	Mean	SD	Mean	SD	*p*	η^2^	power
Number of sessions attended	13.64	4.04	14.48	3.72	0.271	0.01	0.906
Number of relapses	0.75	1.95	0.27	0.69	0.007 *	0.02	0.836

Note. SD: standard deviation. C-V: Cramer-V. η^2^: Partial eta-squared. Observed power (calculated for alpha = 0.05). * Bold: significant comparison. ^†^ Bold: effect size within the ranges moderate to large.

## Data Availability

The data are available upon request from the corresponding author.

## Supplementary Materials

The following supporting information can be downloaded at: https://www.mdpi.com/article/10.3390/jcm11226798/s1, Figure S1. Capacity of the e-Estesia for identifying the risk of relapses (experimental group, *n* = 40). Note. Se: sensibility. Sp: specificity. FAR: false alarm rate; Figure S2. Predictive capacity for the cutoff 8-days use of the e-Estesia (experimental group, *n* = 40). Note. PV+: predictive value for positive screening. PV−: predictive values for negative screening. FDR: false discovery rate; Table S1. Descriptive for the sample; Table S2. Pre-post changes registered for the e-Estesia (experimental group, *n* = 40).
