# Supplementary material for: e-Estesia: A Serious Game for Reducing Arousal, Improving Emotional Regulation and Increasing Wellbeing in Individuals with Gambling Disorder
Source: J Clin Med. 2022 Nov 17;11(22):6798. doi: 10.3390/jcm11226798 (PMC9699009; doi:10.3390/jcm11226798)
Supplement: Supplementary file 1 [file jcm-11-06798-s001.zip › jcm-2034536-supplementary.pdf]

Supplementary Materials

Table S1. Descriptive for the sample.

	Total *n* = 104		Control *n* = 64		Experimental *n* = 40		
	n	%	*n*	%	*n*	%	*p*
Sex							
Women	5	4.8%	1	1.6%	4	10.0%	0.137
Men	99	95.2%	63	98.4%	36	90.0%	
Education							
Primary	54	51.9%	36	56.3%	18	45.0%	0.319
Secondary	40	38.5%	21	32.8%	19	47.5%	
University	10	9.6%	7	10.9%	3	7.5%	
Civil status							
Single	51	49.0%	32	50.0%	19	47.5%	0.969
Married	43	41.3%	26	40.6%	17	42.5%	
Divorced	10	9.6%	6	9.4%	4	10.0%	
Employment							
Unemployed	38	36.5%	16	25.0%	22	55.0%	**0.002** *
Employed	66	63.5%	48	75.0%	18	45.0%	
Social Idex Mean-high to high	8	7.7%	6	9.4%	2	5.0%	0.634
Mean	14	13.5%	8	12.5%	6	15.0%	
Mean-low	38	36.5%	21	32.8%	17	42.5%	
Low	44	42.3%	29	45.3%	15	37.5%	
	**Mean**	**SD**	**Mean**	**SD**	**Mean**	**SD**	*p*
Chronological age (yrs-old)	40.24	14.74	39.56	14.17	41.33	15.73	0.555
Onset of the disorder (yrs-old)	29.66	11.69	28.99	11.28	30.73	12.38	0.464
Duration of the disorder (yrs)	5.31	5.81	5.02	5.57	5.78	6.21	0.519

Note. SD: standard deviation. * Bold: significant comparison.

Table S2. Pre-post changes registered for the e-Estesia (experimental group, *n* = 40).

	Pre-App		Post-App			
	Mean	*SD*	*Mean*	*SD*	*p*	η^2
DERS Non-acceptance	11.88	5.27	11.28	5.29	0.462	0.014
DERS Goals	11.00	4.07	10.03	4.04	0.137	0.056
DERS Impulse	11.25	4.35	10.33	4.23	0.122	0.060
DERS Awareness	18.13	5.28	17.98	5.21	0.854	0.001
DERS Strategy	15.45	6.07	14.13	5.73	0.132	0.057
DERS Clarity	10.30	3.39	9.55	3.68	0.167	0.048
DERS Total score	78.00	18.47	73.28	20.84	0.100	0.068
ERQ Suppression	3.94	1.35	3.36	1.42	**0.021** *	**0.129** †
ERQ Reappraisal	4.13	1.19	3.98	1.32	0.501	0.012
UPPS-P Lack premeditation	23.35	6.03	23.45	5.36	0.877	0.001
UPPS-P Lack perseverance	19.88	4.12	20.25	4.97	0.575	0.008
UPPS-P Sensation seeking	29.05	9.33	29.15	10.55	0.925	0.001
UPPS-P Positive urgency	29.08	9.39	29.05	11.05	0.983	0.001
UPPS-P Negative urgency	29.10	6.66	28.15	8.01	0.264	0.032
UPPS-P Total score	130.45	26.47	130.05	29.89	0.877	0.001
SCL-90R Somatization	0.62	0.73	0.44	0.70	**0.005** *	**0.189** †
SCL-90R Obsessive-compulsive	0.68	0.67	0.41	0.57	**<0.001** *	**0.468** †
SCL-90R Sensitivity	0.69	0.77	0.46	0.71	**0.006** *	**0.179** †

SCL-90R Depression	0.99	0.89	0.60	0.81	**<0.001 ***	**0.301 †**
SCL-90R Anxiety	0.60	0.67	0.33	0.57	**<0.001 ***	**0.284 †**
SCL-90R Hostility	0.44	0.44	0.30	0.53	**0.014 ***	**0.144 †**
SCL-90R Phobic anxiety	0.23	0.42	0.21	0.51	0.696	0.004
SCL-90R Paranoia	0.51	0.55	0.34	0.53	**0.027 ***	**0.119 †**
SCL-90R Psychotic	0.52	0.59	0.36	0.69	**0.005 ***	**0.186 †**
SCL-90R GSI	0.65	0.59	0.43	0.60	**<0.001 ***	**0.355 †**
SCL-90R PST	34.63	21.72	22.78	23.22	**<0.001 ***	**0.403 †**
SCL-90R PSDI	1.49	0.62	1.19	0.67	**0.004 ***	**0.190 †**

Note. SD: standard deviation. * Bold: significant comparison. † Bold: effect size within the ranges moderate to large.

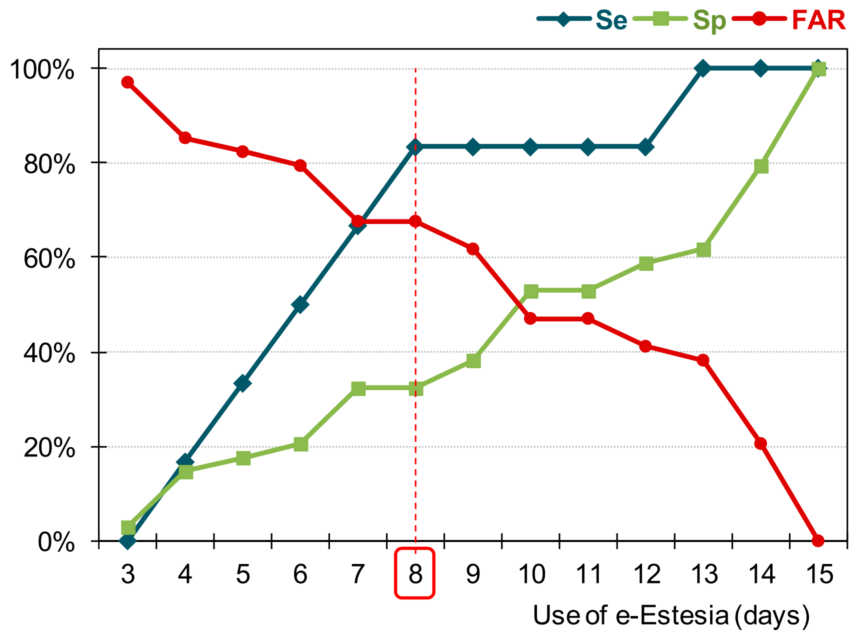

Figure S1. Capacity of the e-Estesia for identifying the risk of relapses (experimental group, *n* = 40). Note. Se: sensibility. Sp: specificity. FAR: false alarm rate.

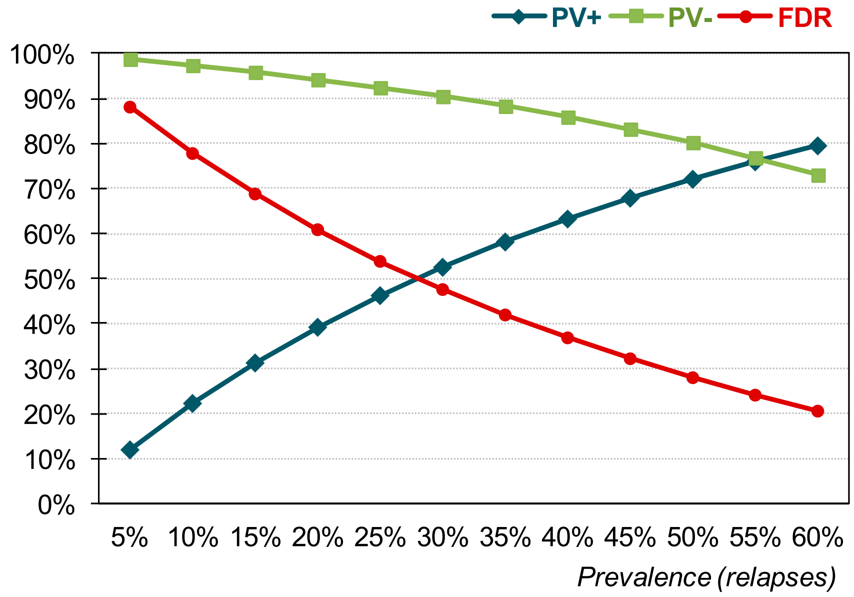

Figure S2. Predictive capacity for the cutoff 8-days use of the e-Estesia (experimental group, *n* = 40). Note. PV+: predictive value for positive screening. PV−: predictive values for negative screening. FDR: false discovery rate.
